# Crystallization Behavior and Electrical Properties of Nanoparticle-Reinforced Poly(lactic Acid)-Based Films

**DOI:** 10.3390/polym14010177

**Published:** 2022-01-02

**Authors:** Mei-Xian Li, Yu Ren, Dasom Lee, Sung-Woong Choi

**Affiliations:** 1 School of Textile and Clothing, Nantong University, Nantong 226019, China; lmx321@ntu.edu.cn (M.-X.L.); ren.y@ntu.edu.cn (Y.R.); 2 Xinfengming Group Huzhou Zhongshi Technology Co., Ltd., Huzhou 313000, China; 3 School of Mechanical and Aerospace Engineering, Seoul National University, Seoul 08826, Korea; dslee07@snu.ac.kr; 4 Department of Mechanical System Engineering, Gyeongsang National University, Tongyeong-si 53064, Korea

**Keywords:** poly(lactic acid), MWNT-Ag, GO, dispersion, electrical conductivity

## Abstract

Graphene oxide (GO) and multiwalled carbon nanotubes with silver particles (MWNT-Ag) of different concentrations were used as nanofillers to prepare poly(lactic acid) (PLA) nanoparticle films through the solvent casting method. In this study, the effects of nanoparticles on the crystallization behavior, relationships between the dispersion and electrical properties, and hydrolytic degradation behaviors were investigated for the PLA/MWNT-Ag and PLA/rGO films. Differential scanning calorimetry was used to evaluate the crystallization behaviors of the PLA/MWNT-Ag and PLA/reduced GO (rGO) films. Electron probe microanalysis was performed to characterize the dispersion of MWNT-Ag, and X-ray diffraction and Raman spectroscopy were used to determine the degree of dispersion of rGO in the PLA matrix. The results showed that nanoparticles enhanced the crystallization kinetics of PLA as well as the hydrolytic degradation rate. From the measurement of electrical properties, the electrical conductivity of PLA/MWNT-Ag 1.0 wt% was much higher than that of the pure PLA and PLA/rGO films, showing that MANT and Ag nanoparticles contribute greatly to enhancing the electrical conductivity of the PLA/MWNT-Ag films.

## 1. Introduction

Poly(lactic acid) (PLA), as one of the environment-friendly biodegradable polymers, has been in the spotlight owing to its excellent mechanical properties [1,2,3,4], and because its hydrolysates are not harmful to the human body or to the environment [5,6,7]. PLA has been used in several industries, such as food packing [8,9], biomedicine [7,10,11,12], and electronics [13,14]. With properties being an issue for PLA, many research groups have made lots of effort to improve the mechanical and electrical conductivity of PLA-based composites by adding various carbon nanofillers, such as multiwalled carbon nanotubes (MWNT), carbon black, graphite, and graphene [1,15,16,17,18,19].

Among these carbon nanofillers, MWNT and graphene are the most popular owing to their superior electrical properties as well as their high aspect ratio. Qian et al. [20] designed PLA-based 3D composites with graphene oxide (GO) nanosheets which were aligned in the filament axial direction. The mechanical properties of these aligned GO nanosheets were outstanding when compared to that of nanosheets with random GO distributions. Dil et al. [21] studied the electrical conductivity of PLA/poly(butylene adipate-co-terephthalate)/MWNT, and their results showed that the MWNTs performed interface bridging, increasing the electrical conductivity of the samples. Guo et al. [22] proposed that a combination of reduced GO (rGO) and graphite could effectively improve the electrical and thermal properties.

Although the addition of nanofillers can enhance various properties of the polymer matrix because of their good mechanical, thermal, and electrical properties, it is difficult to disperse nanofillers in polymer matrixes because of their high aspect ratios. Therefore, dispersion of nanofillers is a crucial parameter in improving the mechanical, thermal, and electrical properties. Regarding the dispersion method for the surface treatment of nanofillers, lots of studies were examined to deal with the dispersion problem using chemical, thermal, and plasma methods. Rong et al. [23] investigated the chemical synthesis of silver nanoparticles by means of microemulsion techniques. Conductive networks can be established by freezing the nanoparticle aggregation. Ma et al. [24] studied the synthesis of MWCNTs using chemical vapor deposition (CVD). They found significant enhancements in dispersion quality and alignment stability for oxidized MWCNTs as compared to pristine MWCNTs. Stankovich et al. [25] examined the reduction of a colloidal suspension of exfoliated graphene oxide sheets in water with hydrazine hydrate. Tiwari et al. [26] has attempted to synthesize metal oxide nanoparticles in the presence of organic reagents, where the hydrothermally synthesized metal oxide nanoparticles can be covered with a hydroxyl group or alkyl chains, which made the surface of the nanoparticles hydrophilic or hydrophobic according to the acquired applications. Allen et al. [27] have undertaken a study on a comparison of the thermal and photochemical behaviors of a selection of nano- versus micron-sized (pigmentary) anatase and rutile titania pigments in polyethylene film and PVC-alkyd, acrylic, and isocyanate-paint films.

To improve the dispersibility of nanofillers in solvents and polymers, various methods have been used to modify their surface chemistry using plasma treatment [28] and plasma oxidation [29] to disentangle the nanofibers. Shi et al. [30] demonstrated the uniform deposition of ultrathin polymer films of 2 nm on the surfaces of Al_2_O_3_ nanoparticles by a plasma treatment. Yu et al. [31] employed plasma treatment method on diamond nanoparticles to improve dispersion in water. They found that the plasma treatment significantly reduced the water-contact angle of diamond nanoparticles and rendered the nanoparticles with a strong water affinity for dispersion enhancement in polar media such as water. Lee et al. [32] has observed plasma treatment of multiwalled carbon nanotubes (MWCNTs) using an atmospheric pressure hydroxyl radical (OH) source with micro-Raman spectroscopy. They found that the dominant effect of OH plasma on MWCNTs was reduced π-conjugated states due to the creation of structural defects and the attachment of oxygen-containing functional groups. Although several studies on the surface treatment of nanofillers using representative chemical, thermal, or plasma methods have been performed [33,34], the data remain insufficient. Moreover, most of the studies with polymer materials focused only on the dispersion phenomenon and results, not on the investigation of the relationship with crystallization behavior.

The objective of our study was to observe and analyze the overall crystallization behavior of PLA/MWNT-Ag and PLA/rGO films with respect to the dispersion behavior of MWNT-Ag and rGO. In this study, the effects of nanoparticles on the crystallization behavior, dispersion, electrical properties, and hydrolytic degradation behavior are investigated for the MWNT-Ag and GO. The crystallization behavior of PLA/MWNT-Ag and PLA/rGO films is evaluated by differential scanning calorimetry (DSC). Electron probe microanalysis (EPMA) is used to characterize the dispersion of MWNT-Ag. The degree of dispersion of rGO in the PLA matrix is determined via X-ray diffraction (XRD) and Raman spectroscopy. Lastly, the electrical conductivity and hydrolytic degradation behavior of PLA/MWNT-Ag and PLA/GO was measured with different concentrations of nanoparticles.

## 2. Experimental Section

### 2.1. Materials

PLA 4032D (NatureWorks, USA; M_n_ ≈ 155,715, M_w_ ≈ 212,921) was used as the matrix in this study. MWNT-Ag (Bioneer, Daejeon, Korea) and GO aqueous solution (Graphene Supermarket, Calverton, USA) were used as conducting nanofillers, and the details of the basic properties of these conducting nanofillers are summarized in Table 1 and Table 2, respectively. Chloroform was used as a solvent to prepare the PLA/MWNT-Ag and PLA/GO films.

### 2.2. Preparation of the PLA/MWNT-Ag Films

**Figure 1:**
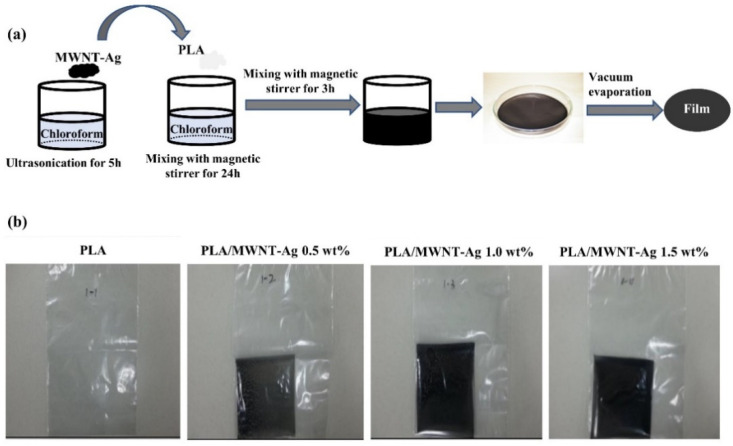
PLA/MWNT-Ag films prepared by solvent casting: (**a**) schematic illustration of the sample preparation; (**b**) PLA/MWNT-Ag film samples with different MWNT-Ag concentrations.

PLA was dissolved in chloroform using a magnetic stirrer with a stirring speed of 200 rpm at room temperature for 24 h, and MWNT-Ag was dispersed in chloroform through bath-type ultrasonication at room temperature for 3 h. During this process, the weight percentage of MWNT-Ag was calculated from that of MWNT (0.5 wt%, 1.0 wt%, and 1.5 wt%) according to the ratio of MWNT and Ag. After that, the MWNT-Ag/chloroform solution was added to the PLA/chloroform solution by mixing with a magnetic stirrer for 3 h to disperse the MWNT-Ag in the PLA solution. The PLA/MWNT-Ag films were created by spreading the solution on a glass Petri dish. Films were exposed to ambient conditions for 24 h, followed by evaporation under vacuum at 60 ℃ for 24 h (Figure 1a). Finally, the PLA/MWNT-Ag film samples with a size of 0.1 m × 0.1 m size were prepared (Figure 1b).

### 2.3. Preparation of the PLA/rGO Films

**Figure 2:**
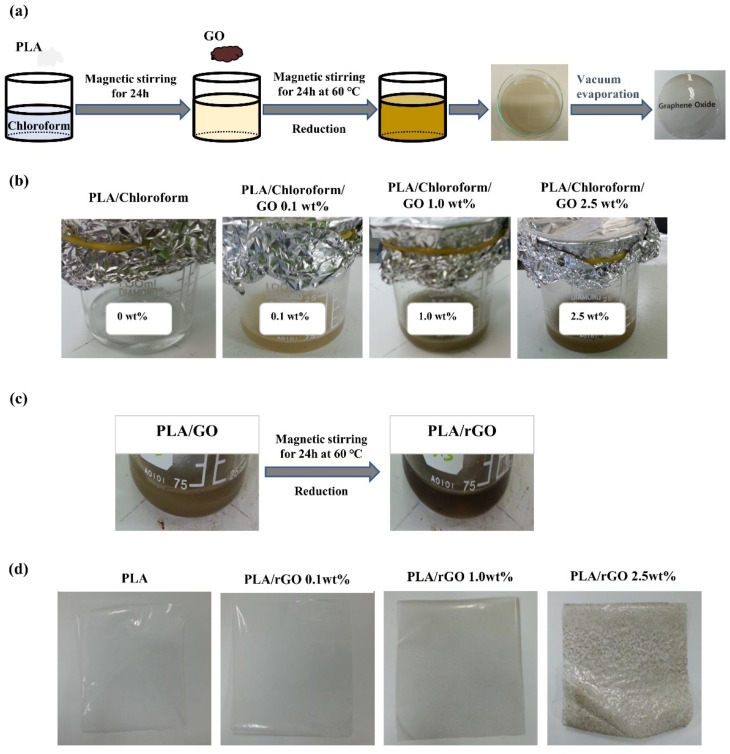
PLA/rGO films prepared by solvent casting: (**a**) schematic illustration of the sample preparation; (**b**) PLA/GO solutions with different GO concentrations; (**c**) color change of PLA/GO solution before and after reduction; and (**d**) PLA/rGO film samples with different rGO concentrations.

PLA was dissolved in chloroform using a magnetic stirrer with a stirring speed of 200 rpm at room temperature for 24 h, followed by adding GO into the PLA/chloroform solution by mixing with a magnetic stirrer at 60 ℃ for 24 h to disperse the GO in the PLA solution. In this step, the color of the GO solution was changed gradually from brown to dark brown according to the GO weight percentage and reduction of GO (Figure 2b,c). The PLA/GO solution was casted on a glass Petri dish, and the solvent was evaporated under vacuum at 60 ℃ for 24 h (Figure 2a). Finally, PLA/rGO film samples of 0.1 m × 0.1 m size were prepared (Figure 2d).

### 2.4. Characterizations

The visual comparison of the transparency for PLA, PLA/rGO, and PLA/MWNT-Ag films was performed. The thermal performance of the PLA-based films was evaluated using DSC (DSC-Q 1000, TA Instrument, New Castle, US) to evaluate the crystallization kinetics and crystallinity. Each film was cut into a small piece weighing approximately 10 mg, and the samples were heated from 30 to 200 ℃ at a rate of 20 ℃/min. The samples were maintained at each temperature for 5 min to erase the previous thermal history. The samples were then cooled down to 110 ℃ and immediately subjected to isothermal scanning at 110 ℃ for 20 min. The samples were subsequently cooled to room temperature as fast as possible. Finally, they were heated again from room temperature to 200 ℃ at a heating rate of 10 ℃/min.

EPMA (JXA-8900R, JEOL, Tokyo, Japan) was performed to examine the degree of dispersion in the PLA/MWNT-Ag films. Samples with a size of 5 mm × 5 mm × 0.03 mm were prepared according to our previous work [35], with a beam diameter of 300 μm × 300 μm. The weight ratio of MWNT and Ag, which are the components of the MWNT-Ag nanoparticles, is 3:7, and the degree of dispersion of MWNT can be estimated from that of the Ag element. In this study, both mapping and quantitative analyses were performed.

XRD analysis (D5005 X-ray diffractometry, Bruker Corporation, Germany) was performed under a voltage of 40 kV and current of 45 mA with Cu Ka radiation (1.5406 Å) with a scanning range of 5 to 30°. The Raman spectra of the samples were measured with a 532 nm wavelength laser (Confocal Micro-Raman Spectrometer, NRS-3100, Japan). The electrical conductivities of the PLA-based films were measured using high-resistance meters (Agilent, 4339B, Santa Clara, USA) with an applied voltage of 100 V (according to ASTM D257).

The degradation behaviors of the samples were evaluated according to our previously reported method [36]. The samples were immersed in a sodium hydroxide (NaOH) solution (PH = 13) at 37 ℃ for a predetermined period, and then rinsed with distilled water until the pH approached 7. Finally, the samples were dried in a vacuum oven at 70 ℃ for 48 h. The weight loss (*W*_loss_) was estimated using the following equation:

(1) $$W_{\mathrm{loss}}(\%)=100\%\times (W_{0}-W_{t})/W_{0}$$

where *W*_0_ is the initial weight of the specimen, *W*_t_ is the weight of the specimen dried in a vacuum oven at 70 ℃ for 48 h, and *W*_loss_ is the weight loss of the specimen.

## 3. Results and Discussion

### 3.1. Transparency

**Figure 3:**
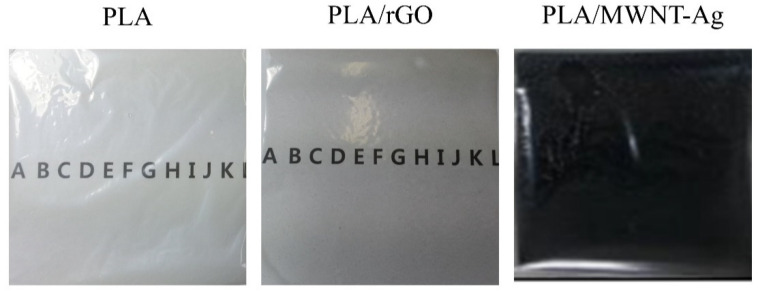
Transparency of PLA, PLA/rGO 1.0 wt%, and PLA/MWNT-Ag 1.0 wt% films.

All PLA/MWNT-Ag films were opaque with a low-weight percentage of MWNT-Ag (Figure 1b), whereas, the PLA/rGO films were transparent at lower rGO concentrations (Figure 2d). However, the PLA/rGO films became slightly opaque when the concentration of rGO was higher than 2.5 wt%, owing to the uneven dispersion of the rGO particles. As shown in Figure 3, a highly transparent sample of the PLA/rGO 1.0 wt% film was observed, similar to the PLA film.

### 3.2. DSC Characterization

**Figure 4:**
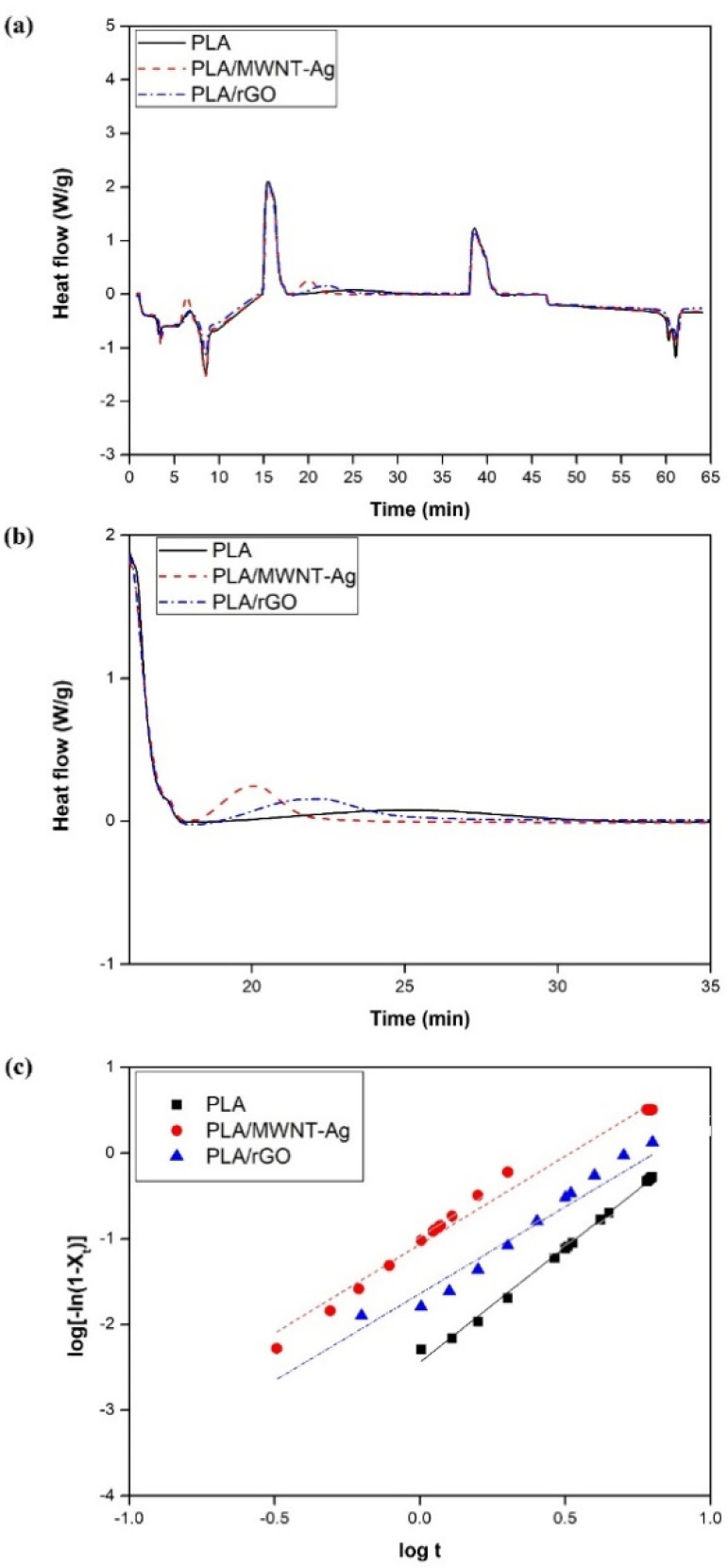
DSC thermograms for samples with different types of nanoparticles: (**a**) results of heating–cooling–heating cycles for the crystallization behavior of PLA-based films; (**b**) isothermal DSC thermograms at 110 ℃; and (**c**) plots of log [ln(1−*X*_t_)] versus log *t* for the PLA-based films at 110 ℃.

The crystallization kinetics of the PLA-based films were evaluated. Figure 4a shows the overall heating–cooling–heating cycles for the crystallization behaviors of the PLA, PLA/MWNT-Ag, and PLA/rGO films. The isothermal scanning results of the PLA-based films are presented in Figure 4b, and it can be observed that it takes more than 15 min to fully crystallize pure PLA, while the crystallization time is reduced by almost one third for the PLA/MWNT-Ag and PLA/rGO films. The crystallization rate increases with the addition of nanoparticles, which can react as nucleation agents. The Avrami equation was used to evaluate the isothermal crystallization mechanisms of the PLA, PLA/MWNT-Ag, and PLA/rGO films. The relative crystallinity could be calculated from Equation (2):

(2) $$X_{t}=\frac{\int_{0}^{t}(dH/dt)dt}{\int_{0}^{\infty }(dH/dt)dt}$$

where d*H*/d*t* is the respective heat flow; the sum of d*H*/d*t* from 0 to *t* is the enthalpy at time *t*; and the sum of d*H*/d*t* from 0 to *t*_∞_ is the enthalpy at time *t*_∞_, which can be obtained as the total area under the crystallization DSC curve. Generally, the Avrami equation can be converted to the following linear form:

(3) $$\log[-\ln(1-X_{t})]=\log K+n\log t$$

where *n* is the Avrami exponent that reflects the nucleation mechanism and dimension of crystal growth [37,38]. The temperature-dependent parameter *K* is the kinetic rate constant. By plotting the Avrami equation, log [ln(1−*X*_t_)] versus log t, the values of *n* and *K* can be obtained from the intercept and slope, respectively (Figure 4c).

The crystallization half-time (*t*_1/2_), which is the time required to reach 50% crystallinity and indicates the crystallization rate, can be defined by Equation (4).

(4) $$t_{1/2}={\left(\ln2/K\right)}^{1/n}$$

The thermal characteristic parameters such as the crystallization temperature (*T*_c_), glass transition temperature (*T*_g_), melting temperature (*T*_m_), n, K, and *t*_1/2_, are listed in Table 3.

For the degree of dispersion, XRD and Raman spectroscopy were used to evaluate the dispersion degree for the PLA/rGO, and EPMA was used for the PLA/MWNT-Ag with different concentration of 0.5–1.5 wt.%. Among them, a concentration of 1.0 wt.% was found to be well dispersed. Therefore, among the samples with different nanoparticle concentrations, PLA, PLA/MWNT-Ag 1.0 wt%, and PLA/rGO 1.0 wt% samples were prepared. The samples were dispersed to evaluate their crystallization kinetics from a DSC thermogram. The results show that the glass transition temperature and melting temperature of the PLA-based films remained almost same, without being affected by the nanoparticles, while the crystallization rate increased significantly with the addition of nanoparticles that acted as nucleating agents. Meanwhile, the crystallization rate of PLA/MWNT-Ag was faster than that of PLA/rGO. The reason could be that both MWNT and Ag nanoparticles acted as nucleating agents and affected the crystal formation and growth. Avrami exponent values of *n* for pure PLA, PLA/MWNT-Ag, and PLA/rGO were observed in the vicinity of 2, indicating that the crystallization mechanism was two-dimensional growth. Therefore, the addition of MWNT-Ag or rGO into the PLA matrix did not affect the geometric dimensions of PLA crystal growth during isothermal crystallization.

With the increasing of the concentration of the nanoparticles, the isothermal crystallization rate was found to be increased. However, after some point, the tendency to increase was reduced. This is because too many nanoparticles could hinder crystal growth. In sum, the crystallization rate was increased in early time with increasing concentrations of the nanoparticles. After some point, the crystallization rate was shown to decrease.

### 3.3. Dispersion of Nanoparticles in PLA

Both EPMA mapping and quantitative analysis were performed to evaluate the degree of dispersion of the MWNT-Ag nanoparticles in the PLA matrix. The degree of dispersion of MWNT could be estimated from that of the Ag elements, because of the grafting of Ag on MWNT.

**Figure 5:**
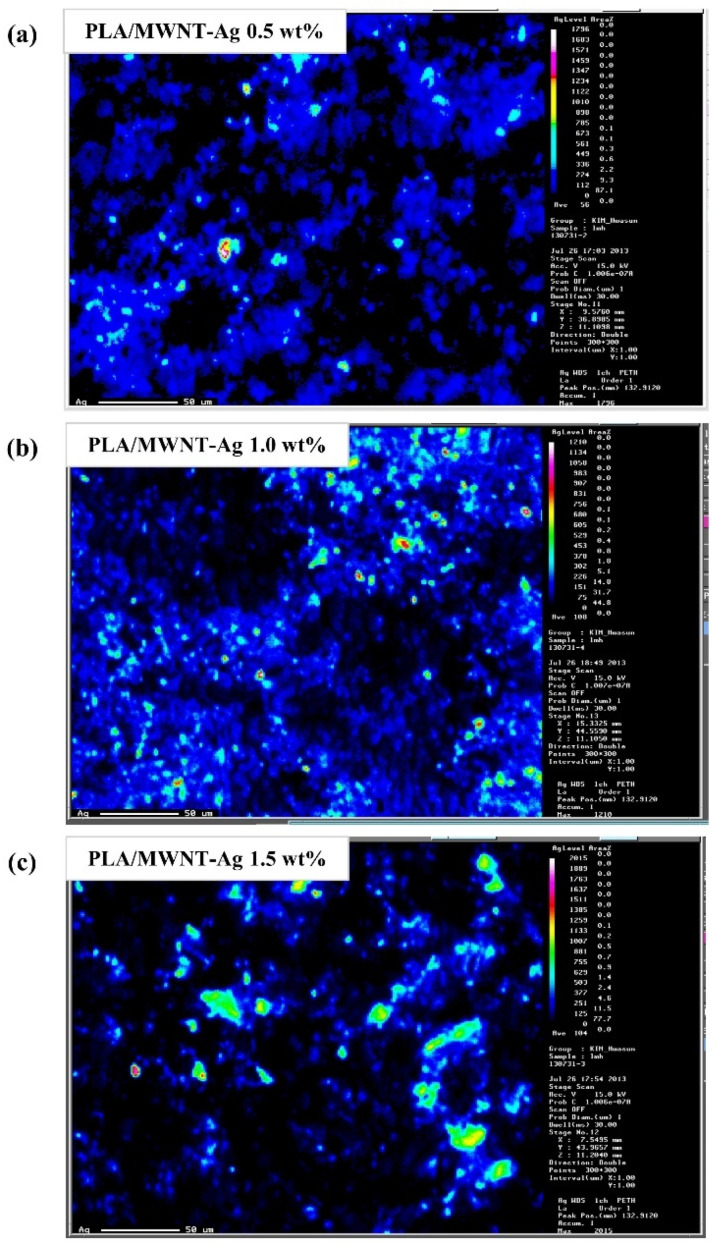
EPMA mapping images of PLA/MWNT-Ag films: (**a**) PLA/MWNT-Ag 0.5 wt%; (**b**) PLA/MWNT-Ag 1.0 wt%; and (**c**) PLA/MWNT-Ag 1.5 wt%.

Figure 5 shows the EPMA mapping images of PLA/MWNT-Ag films with different concentrations of MWNT-Ag nanoparticles. Ag is observed to be relatively well dispersed in PLA/MWNT-Ag films with 0.5 wt% and 1.0 wt% MWNT-Ag, especially. On the other hand, there are some distinct aggregations in the PLA/MWNT-Ag films with 1.5 wt% MWNT-Ag.

**Figure 6:**
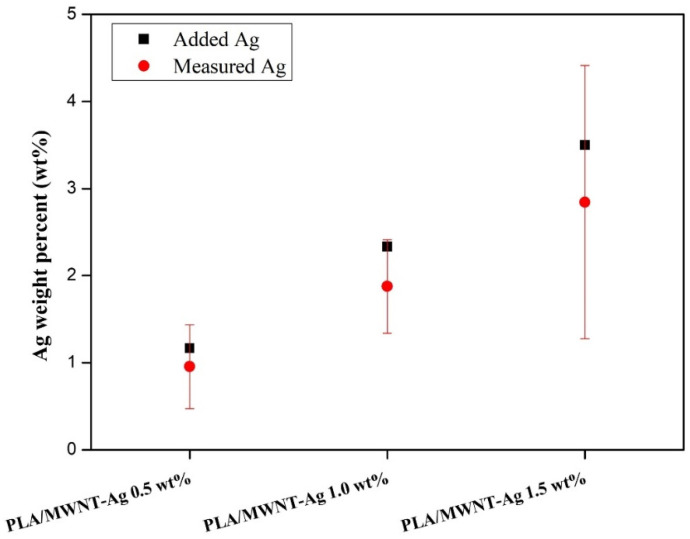
EPMA quantitative analysis of PLA/MWNT-Ag films.

Quantitative analysis was conducted with the same samples in order to measure the degree of dispersion more precisely. In Figure 6, the black dot indicates the weight percentage of Ag added to the PLA matrix, while the red dot represents that of Ag measured by the EPMA equipment. The measured weight percentage of Ag is slightly lower than the added Ag weight percentage. The reason may be that there still exist some aggregation parts of MWNT-Ag after 24 h sonication. Additionally, the relatively wide range of standard deviations is due to more nanoparticle aggregation.

For the PLA/rGO samples, it is difficult to evaluate the degree of dispersion of rGO using EPMA because both PLA and rGO are composed of only C, H, and O elements. Therefore, to roughly estimate the degree of dispersion of rGO in the PLA matrix, measurements using XRD and Raman spectroscopy were conducted. PLA with 1.0 wt% rGO film did not show the characteristic peak of rGO, indicating that rGO was relatively well dispersed in the PLA matrix.

**Figure 7:**
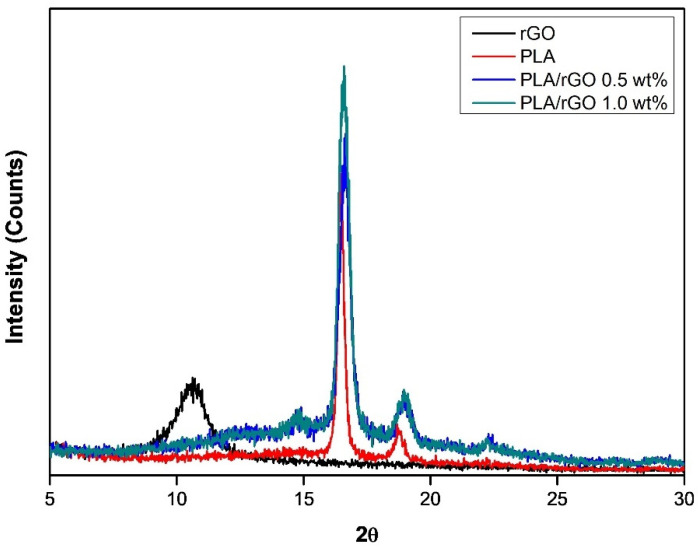
XRD patterns of rGO, pure PLA, PLA/rGO 0.5wt%, and PLA/rGO 1.0 wt%.

The XRD patterns of pure rGO, pure PLA, PLA/rGO 0.5 wt%, and PLA/rGO 1.0 wt% are shown in Figure 7. Pure PLA exhibits two sharp peaks at 2θ = 16.58° and 18.95°, which reflects crystal planes (200)/(110) and (203) indicating α- and α’- crystal formation. These results are in accordance with the results of a study by Kalani and Yunus [39]. As can be seen in Figure 7, there is a strong peak around 10.63°, which corresponds to the rGO structure. However, the peak associated with rGO disappeared for the PLA/rGO 0.5 wt% and PLA/rGO 1.0 wt% samples. It could be inferred that rGO was evenly dispersed in the PLA matrix. Meanwhile, the peaks around 16.58° and 18.95° for the PLA/rGO samples were more prominent than those of pure PLA, indicating more crystallinity owing to the rGO acting as a nucleating agent.

**Figure 8:**
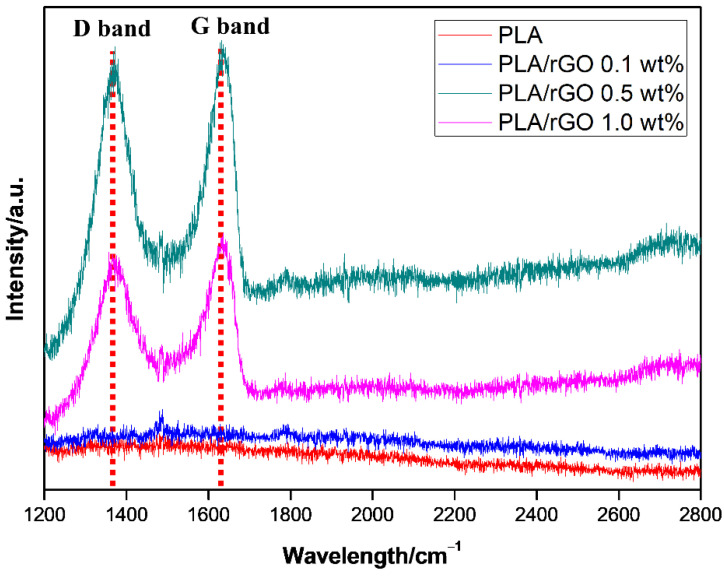
Raman spectra of PLA/rGO 0.5 wt% and PLA/rGO 1.0 wt%.

The Raman spectra of PLA/rGO 0.5 wt% and PLA/rGO 1.0 wt% are shown in Figure 8. The intensity of the D band (around 1400 cm^−1^) and G band (around 1600 cm^−1^) corresponded to the vibrations of the sp^3^-bonded carbon atoms and sp^2^-bonded carbon atoms, respectively. As already known, the D band reflects disordered structures (such as defects, crystal boundaries, and so on), while the G band reflects the crystalline structures [40]. The intensity ratios of the D band and G band (*I*_D_/*I*_G_) for PLA/rGO 0.5 wt% and PLA/rGO 1.0 wt% were 0.93 and 0.99, respectively, which were significantly higher than that of pure graphite [41], because GO was successfully exfoliated after reduction. In addition, there was no 2D band between 2600 cm^−1^ and 2800 cm^−1^, indicating no stacked graphene layers.

### 3.4. Electrical Properties

**Figure 9:**
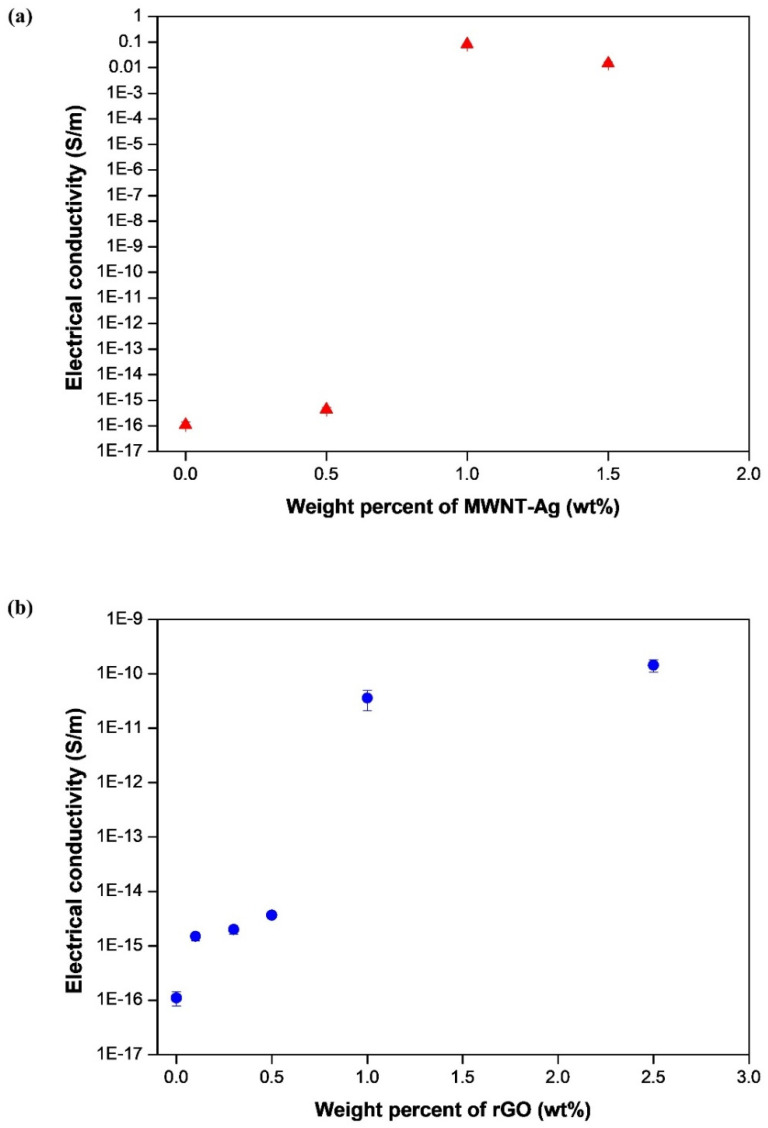
Electrical conductivity of: (**a**) PLA/MWNT-Ag; (**b**) PLA/rGO films.

The electrical properties were analyzed for different concentrations of MWNT-Ag and/or rGO (Figure 9). The results showed that the electrical conductivity of PLA/MWNT-Ag and PLA/GO increased as the concentration of nanoparticles increased. PLA films with more than 1.0 wt% nanoparticles showed significantly higher electrical conductivities than pure PLA and PLA films with less than 0.5wt% nanoparticles. In addition, the electrical conductivity of PLA/MWNT-Ag 1.0wt%, which exhibited the best dispersion, was shown to be remarkably higher than that of pure PLA and other PLA films. Finally, PLA/MWNT-Ag films showed much higher electrical conductivities than PLA/rGO films for the same nanoparticle concentration. Ag nanoparticles also contributed greatly to enhancing the electrical conductivity of the samples; therefore, relatively higher electrical conductivities were observed with PLA/MWNT-Ag films.

### 3.5. Degradation Properties

**Figure 10:**
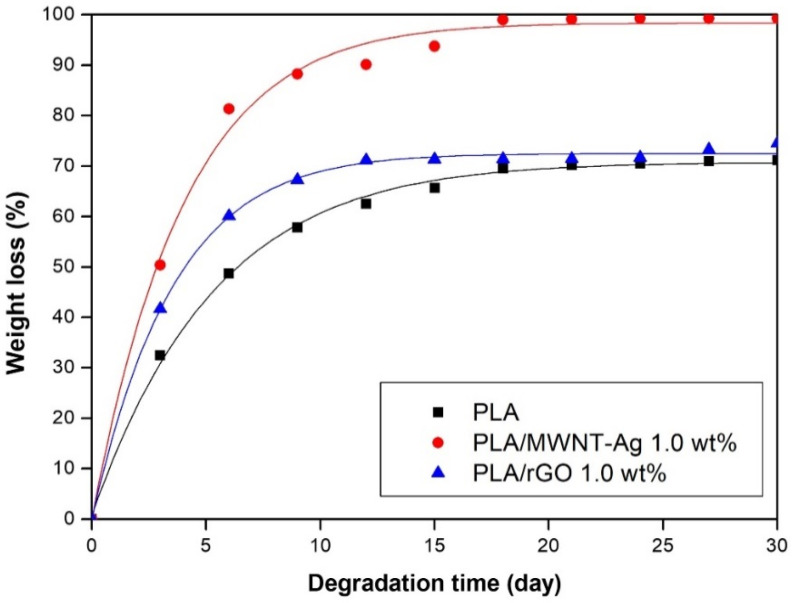
Degradation of PLA-based films.

The weight losses of pure PLA, PLA/MWNT-Ag, and PLA/rGO with degradation time are shown in Figure 10. The weight losses of all samples exceeded 50% within 10 days, and then, the degradation rate was decreased. The degradation rates of PLA/MWNT-Ag and PLA/rGO were higher than that of pure PLA because of the interface between the particles and the PLA matrix, which served as a leading path for hydrolytic degradation. In this study, the same weight percentage of MWNT and rGO were used in the PLA matrix. However, the Ag nanoparticles were grafted on MWNT; thus, the total weight percentage of the nanoparticles for the PLA/MWNT-Ag was higher than that of PLA/rGO. It means that the surface areas of the interfaces of PLA/MWNT-Ag were much larger than that of PLA/rGO. Therefore, the degradation rate of PLA/MWNT-Ag was higher than that of PLA/rGO. Another perspective of the above phenomenon was that increasing the crystallinity of the samples was attributed to making it more difficult to degrade parts than the amorphous parts for the PLA/rGO films prepared at 60 ℃. Therefore, the degradation rate of PLA/rGO decreases compared to that of PLA/MWNT-Ag. These observed tendencies were identical to those of our previous study [42].

Overall, transparent and flexible PLA/rGO thin films were prepared via a solvent casting method which overcame the brittle samples prepared in previous works. PLA/MWNT-Ag thin films prepared in this work were flexible and had high electrical conductivity. In this study quantitative and qualitative assessment could be conducted at the same time due to the Ag nanoparticles grafted on the MWNT which could easily evaluate the distribution of nanoparticles visually comparted to other research.

## 4. Conclusions

In this study, PLA/MWNT-Ag and PLA/rGO films were prepared to investigate the effects of nanoparticles on crystallization behavior, dispersion, electrical conductivity, and hydrolytic degradation behavior. For the PLA-based film of PLA/MWNT-Ag and PLA/rGO, there was no significant difference between the glass transition temperature and the melting temperature, while the crystallization rate increased significantly with the addition of nanoparticles that acted as nucleating agents. The crystallization rate of PLA/MWNT-Ag was faster than that of PLA/rGO. The results showed that nanoparticles such as MWNT-Ag and rGO acted as nucleating agents to enhance the crystallization rate and crystallinity of the samples. From the XRD patterns of pure rGO, pure PLA, PLA/rGO 0.5 wt%, and PLA/rGO 1.0 wt%, PLA/rGO was shown to have more crystallinity, owing to the rGO acting as a nucleating agent. The electrical conductivity of PLA/MWNT-Ag and PLA/GO increased as the concentration of nanoparticles increased. The electrical properties of the PLA films were affected by the nanoparticles and their dispersion degrees. At the same nanoparticle concentration, the electrical conductivity of PLA/MWNT-Ag was much higher than that of PLA/rGO, because Ag nanoparticles also contribute greatly to enhancing the electrical conductivity of the samples. The degradation rates of PLA/MWNT-Ag and PLA/rGO were higher than that of pure PLA because of the interface between the particles and the PLA matrix, which served as a leading path for hydrolytic degradation. The degradation rate of PLA/MWNT-Ag was also higher than that of PLA/rGO because of the larger surface areas of the interface of PLA/MWNT-Ag, compared to that of PLA/rGO. As GO has good transparency, it can be utilized to make biodegradable polymer-based thin films with high transparency and high electrical conductivity. The results and method presented in this paper can trigger further research endeavors in the direction of fabricating transparent biodegradable films with high electrical conductivity.

## Figures and Tables

**Table 1:** Features of MWNT-Ag.

Property	MWNT-Ag Filler
Mass ratio	MWNT:Ag = 3:7
Average diameter of Ag (nm)	100
Diameter of MWNT (nm)	10–15
Length of MWNT (nm)	10–20

**Table 2:** Features of GO aqueous solution.

Property	MWNT-Ag Filler
Composition of carbon and oxygen (%)	Carbon (79), Oxygen (20)
Flake size (μm)	0.5–5
Thickness	1 atomic layer—at least 60%
Color	Brown
Concentration (g/L)	6.2 (Aqueous GO)
Single layer (%)	>80

**Table 3:** Thermal characteristic parameters of PLA-based films, obtained from DSC.

	*T*_c_ (℃)	*T*_g_ (℃)	*T*_m_ (℃)	*n*	*K* (min−n)	*t*_1/2_ (min)
PLA	110	64	171	2.18	0.007	8.28
PLA/MWNT-Ag	110	64	171	2.19	0.105	2.37
PLA/rGO	110	64	171	2.07	0.023	5.13

## Data Availability

The data presented in this study are available on request from the corresponding author.
